# Soot and Smoke

**Published:** 1907-12-28

**Authors:** 


### Soot and Smoke.

The harmful effects of London fogs on vegetation have been demonstrated time after time, and the fact that it is impossible for an evergreen to exist in the metropolis is a striking proof of the vitiated atmosphere in which most of us have to live and work. Medical men are familiar with the appearance of the lungs shown at post-mortem examinations of those who are residents in a sooty atmosphere; and although there is a tendency to regard such thundercloud-blue lungs as not being definitely pathological, it is certainly reasonable to suppose that individuals whose pulmonary tissue is loaded with such material impurity and whose bronchial glands are choked with fog pigment must be less able to withstand the effects of exposure to infectious diseases. We are aware that, to quote one specific instance, it is alleged that fog-bound individuals, dwellers in large cities, are less liable to pulmonary phthisis, but that allegation has by no means been proved. Little by little our knowledge of the incidence of consumption is getting broadened, and not always along lines which we might have expected from what has hitherto been known concerning the disease. Nor is it impossible that we shall have in the future to modify very seriously some of our generally accepted conclusions with regard to the etiology of this plague of civilisation. Professor Karl Pearson's statistics, however fragmentary and incomplete they may be, are yet sufficiently interesting to make us pause before accepting as proved facts the old text-book dicta about the incidence of phthisis. In the circumstances it is desirable that someone should reconsider this question of the liability of city dwellers to tubercular disease, and we venture to think that, when such an investigation is carried out systematically and on strict actuarial principles, it will be found that the incidence of consumption is much greater among those exposed to the sulphuric-acid fogs of the larger manufacturing towns than it is among those whose lives lie in cleaner and more sanitary surroundings. Nor is it only upon the living that the fog works havoc. Our public buildings, our national monuments, our waterways, and our pleasure-grounds are alike annually damaged to a greater or less extent by an evil which, by the outlay of some capital and the expenditure of some care, would be largely minimised, if not wholly subdued. What use is it to preach the doctrine of fresh air and open windows when the only air to be obtained is so laden with impurities that it is imperative to shut it out from the dwelling-room? During the continuance of a fog the atmosphere in our churches, public libraries, and large commercial establishments is so close, unwholesome, and impure that it must of necessity create all those evils which we are accustomed to tabulate as the effects of a vitiated atmosphere.

No words can be strong enough to condemn the apathy of the public authorities who permit the smoke nuisance to continue unabated, although they have the power, by law, to check it. The manufacturer who permits his chimney to belch forth tons of diluted sulphuric acid is a fool. At the expenditure of a comparatively small capital outlay he can fit his furnaces with smoke-combustion apparatus, which, by diminishing the coal bill, will in the course of a few years save more than the initial outlay necessary for its erection. But if the manufacturer is at liberty to allow a part of his fuel to run to waste, he is certainly not entitled to allow that wastage to become a public nuisance, thereby endangering the health and the property of his neighbours. There is a law to compel him to take certain precautions, which, if seriously carried out, will largely prevent our atmosphere from being turned into that of a coal mine, and it is imperative that this law should be strictly enforced. It is so enforced in some provincial towns. Why, then are its clauses neglected in London and Manchester? To take one instance, during the past few years no less than 1,788 complaints—specific, detailed, and worthy of attention—were made to the West Ham Corporation. Not one of these was properly considered, and the Local Government Board declined to interfere. It is only when such reports are actively followed up, and the legal enactments for the prevention of the smoke nuisance better enforced, that London fogs will be less frequent and less severe, and the nation will save some thousands of lives and many thousands of pounds.

